# Dental Pulp Stem Cell-Derived Small Extracellular Vesicle miR-100-5p Attenuates Neuronal Oxidative Stress via Regulating the mTOR-Autophagy Signaling Axis

**DOI:** 10.3390/cells15201843

**Published:** 2026-10-11

**Authors:** Wenli Zhou, Yanfang Zhao, Fengtang Yang, Wensheng Zhang, Xiuzhen Zhang

**Affiliations:** 1 School of Life Sciences and Medicine, Shandong University of Technology, 266 Xincun West Road, Zhangdian, Zibo 255049, China; 23410010997@stumail.sdut.edu.cn (W.Z.); zhaoyanfang1@126.com (Y.Z.); fengtangyang@163.com (F.Y.); 2 State Key Laboratory of New Targets Discovery and Drug Development for Major Diseases, Gannan Innovation and Translational Medicine Research Institute, Gannan Medical University, Ganzhou 341000, China

**Keywords:** small extracellular vesicles, dental pulp stem cells, oxidative stress, microRNA, autophagy, mTOR

## Abstract

**Highlights:**

**What are the main findings?**

- Small extracellular vesicles (sEVs) derived from human dental pulp stem cells (hDPSCs) exert robust protective effects against H_2_O_2_-induced oxidative injury SH-SY5Y neuronal cells.
- miR-100-5p enriched in hDPSC-sEVs confers neuroprotective and antioxidant activities, likely via mTOR suppression and subsequent autophagy activation in SH-SY5Y cells.

**What are the implications of the main findings?**

- miR-100-5p is identified as a key functional effector mediating the in vitro neuroprotective capacity of hDPSC-sEVs.
- These findings provide preliminary evidence supporting hDPSC-sEVs as a novel cell-free antioxidant therapeutic candidate for neural damage.

**Abstract:**

Excessive accumulation of reactive oxygen species (ROS) in the brain and the resulting oxidative stress are core drivers of neurodegenerative processes. Human dental pulp stem cells (DPSCs) secrete small extracellular vesicles (sEVs) abundant in functional microRNAs, which possess prominent regenerative capacity. The present study investigated the neuroprotective effects of DPSC-derived sEVs (DPSC-sEVs) against oxidative injury in SH-SY5Y neuronal cells and elucidated the underlying molecular mechanisms. DPSC identity was confirmed via flow cytometric immunophenotyping and multipotent differentiation assays. sEVs were isolated from DPSC-conditioned medium and characterized by transmission electron microscopy, nanoparticle tracking analysis and detection of canonical exosomal markers (CD63, CD81, and TSG101). An in vitro model of neuronal oxidative stress was established using hydrogen peroxide (H_2_O_2_). Cell viability, apoptosis, intracellular ROS levels, and lactate dehydrogenase (LDH) release, as well as the levels/activities of superoxide dismutase (SOD), glutathione peroxidase (GSH-Px) and malondialdehyde (MDA), were assessed to evaluate the protective effects of sEVs. Candidate miRNAs in sEVs were screened by quantitative real-time PCR (qPCR). Direct binding of miR-100-5p to the 3′ untranslated region (3′UTR) of mammalian target of rapamycin (mTOR) was verified via dual-luciferase reporter assay. Autophagic activity was evaluated by immunoblotting and immunofluorescence staining of LC3B/p62. Pretreatment with DPSC-sEVs significantly ameliorated H_2_O_2_-induced neuronal oxidative damage and apoptosis. Mechanistically, sEV miR-100-5p conferred neuroprotective and antioxidant properties by inhibiting mTOR signaling and activating autophagy in vitro. Blockade of miR-100-5p or pharmacological inhibition of autophagy by 3-methyladenine (3-MA) largely abolished these protective effects. In conclusion, DPSC-sEVs attenuate neuronal oxidative stress by reversing mTOR-mediated suppression of autophagy, and this protective effect is dependent on the function of miR-100-5p in SH-SY5Y cells. The miR-100-5p/mTOR/autophagy axis mediates the neuroprotective effects of DPSC-sEVs, representing a promising therapeutic candidate for oxidative stress-associated neurodegenerative diseases that merits further in vivo validation.

## 1. Introduction

Oxidative stress refers to a state where the production of reactive oxygen species (ROS) and reactive nitrogen species (RNS) in the body overwhelms the scavenging capacity of the endogenous antioxidant defense system. This imbalance between oxidative and antioxidant systems drives excessive accumulation of highly reactive intermediates, which ultimately results in tissue damage [1]. The brain is particularly vulnerable to oxidative stress owing to its high lipid content, which renders it prone to ROS generation. Accumulating evidence indicates that ROS play a pivotal role in both physiological brain function and pathological processes; disruption of redox homeostasis directly contributes to neuronal injury and neurological dysfunction [2].

In stem cell research, multipotent stem cell populations have garnered widespread attention for their therapeutic potential. Among these, dental pulp-derived stem cells stand out as a particularly promising candidate due to their unique advantages [3]. Similar to other mesenchymal stem cells (MSCs), dental pulp stem cells (DPSCs) exhibit multipotent differentiation capacity, self-renewal capability, and robust proliferative ability in response to microenvironmental cues. These cells can differentiate into odontoblasts, osteoblasts, chondrocytes, adipocytes, and neural lineage cells, thereby contributing to tissue repair and regeneration processes [4]. Compared with other MSC sources, DPSCs are more readily accessible for isolation and possess low immunogenicity, which reduces the risk of immune rejection after transplantation [5]. Accumulating evidence shows that neurotrophic factors mediate neuroprotection and underpin the therapeutic benefits of cell-based therapies for neurodegenerative diseases [6]. Notably, the expression level of neurotrophic factors in DPSCs is higher than that in human bone marrow-derived MSCs and human adipose-derived MSCs, further highlighting the substantial potential of DPSCs for treating neurological disorders [7].

Small extracellular vesicles (sEVs) are nanosized membrane vesicles secreted by cells, representing a cell-free therapeutic strategy that circumvents the limitations of conventional cell therapy [8]. sEVs are a subset of extracellular vesicles with an diameter of approximate 100 nm, enclosed by a lipid bilayer [9]. Their cargo includes proteins, lipids, DNA, and diverse RNA species such as messenger RNA (mRNA), long non-coding RNA (lncRNA), and microRNA (miRNA) [10,11,12]. sEVs have been shown to mediate a broad range of biological functions, including antigen presentation, intercellular communication, and immune regulation [13,14,15].

miRNAs are a class of small non-coding RNAs of 18–24 nucleotides that regulate gene expression at the post-transcriptional level [16]. As highly abundant non-coding RNA in the brain, miRNAs are closely implicated in the regulation of neurodevelopment, the maintenance of synaptic plasticity, and the progression of neurodegenerative diseases [17,18]. Previous studies have demonstrated that DPSC-derived sEVs (DPSC-sEVs) are enriched in miR-100-5p [19], a microRNA that plays a critical role in modulating cellular metabolism, inflammatory responses, and tissue regeneration by targeting and inhibiting mammalian target of rapamycin (mTOR) [20,21,22,23]. mTOR is well established as a key negative regulator of autophagy: activation of mTOR suppresses autophagic flux, whereas inhibition of mTOR promotes autophagy [24,25].

Autophagy is an essential homeostatic mechanism that maintains cellular integrity by selectively degrading damaged or redundant cellular components [26]. It is recognized as a critical cellular defense mechanism against oxidative stress and other insults that cause the accumulation of damaged proteins or organelles [27]. Accumulating studies have revealed that stem cell-derived sEVs exert cytoprotective effects by modulating apoptosis and inflammatory processes via autophagy activation [28,29,30]. For example, human umbilical cord MSC-derived sEVs alleviate corneal injury by inducing autophagy [28]; MSC-derived sEVs rescue pathological phenotypes in Parkinson’s disease models through autophagy induction [29]; and bone marrow MSC-derived sEVs improve functional recovery after spinal cord injury in rats by activating autophagy [30]. In this study, we hypothesized that DPSC-sEVs protect SH-SY5Y cells from oxidative damage by delivering miR-100-5p to modulate the mTOR pathway and subsequently regulate autophagy.

## 2. Materials and Methods

### 2.1. Cell Culture

Human dental pulp stem cells (hDPSCs) were kindly provided by Shandong Shan yuan Biotechnology Co., Ltd. (Ji’nan, China) Ethical approval and written informed consent for the use of these cells were obtained by the supplier. DPSCs were cultured in α-minimum essential medium (α-MEM; Shandong Qidu Pharmaceutical Co., Ltd., Zibo, Shandong, China) supplemented with 10% fetal bovine serum (FBS; VivaCell, XP Biomed Ltd., Shanghai, China).

The human neuroblastoma SH-SY5Y cell line was purchased from the Cell Bank of the Chinese Academy of Sciences (National Resource Library of Model & Characteristic Experimental Cell Resources, Center for Excellence in Molecular Cell Science, Chinese Academy of Sciences, Shanghai, China). SH-SY5Y cells were maintained in Dulbecco’s modified Eagle’s medium/Nutrient Mixture F-12 (DMEM/F12 medium; Gibco, Thermo Fisher Scientific (China) Co., Ltd., Suzhou, China) containing 10% FBS, 100 U/mL penicillin, and 100 µg/mL streptomycin (all from Viva Cell, XP Biomed Ltd., Shanghai, China). All cells were incubated at 37 °C in a humidified atmosphere with 5% CO_2_ using a HERACELL 250i incubator (Thermo Fisher Scientific, Langenselbold, Germany).

### 2.2. Identification of DPSCs

Immunophenotypic identification of DPSCs was performed by flow cytometry to confirm positive expression of mesenchymal stem cell markers CD73, CD90, and CD105 and negative expression of hematopoietic markers CD45, CD14, CD34, CD19, and HLA-DR (all antibodies from Tianjin Haoyang Huake Biotechnology Co., Ltd., Tianjin, China).

To evaluate multilineage differentiation capacity, DPSCs were cultured in osteogenic or chondrogenic induction medium for 3 weeks. Osteogenic induction medium consisted of α-MEM containing 10%FBS, 100 U/mL penicillin, 100 μg/mL streptomycin, 10 nM dexamethasone (Solarbio, Beijing, China), 50 μg/mL L-ascorbic acid-2-phosphate (Solarbio, Beijing, China), and 10 mM β-glycerophosphate (Solarbio, Beijing, China). Chondrogenic induction medium was based on high glucose DMEM (4.5 g/L glucose) (Gibco, Thermo Fisher Scientific (China) Co., Ltd., Suzhou, China) supplemented with 100 U/mL penicillin, 100 μg/mL streptomycin, 1% (*v*/*v*) ITS-Premix (Gibco, Thermo Fisher Scientific (China) Co., Ltd., Suzhou, China), 100 nM dexamethasone (Solarbio, Beijing, China), 50 μg/mL L-ascorbic acid-2-phosphate (Solarbio, Beijing, China), 40 μg/mL L-proline (Solarbio, Beijing, China), 1 mM sodium pyruvate (Solarbio, Beijing, China), 1% (*v*/*v*) non-essential amino acids (NEAAs; Gibco, Thermo Fisher Scientific (China) Co., Ltd., Suzhou, China) and 10 ng/mL recombinant human transforming growth factor-β3 (TGF-β3) (PeproTech, Rocky Hill, NJ, USA).

Mineralized nodule formation and proteoglycan deposition were detected by Alizarin Red S staining and Alcian Blue staining (Solarbio, Beijing, China), respectively. Each experiment included three technical replicates per group, and all results were verified in at least three independent biological experiments using different cell passages.

### 2.3. Isolation of sEVs

sEVs were isolated from DPSC-conditioned medium via a polyethylene glycol (PEG)-based precipitation method. Briefly, DPSCs were cultured in serum-free α-MEM, and the culture supernatant was collected after appropriate incubation. The supernatant was sequentially centrifuged at 300× *g* for 10 min and 2000× *g* for 10 min at 4 °C to remove intact cells and cell debris. The clarified supernatant was filtered through a 0.22 µm filter to eliminate larger particles, then thoroughly mixed with 40% (*w*/*v*) PEG 6000 (Sangon Biotech (Shanghai) Co., Ltd., Shanghai, China) solution at a volume ratio of 4:1, yielding a final PEG concentration of 8%. The mixture was incubated overnight at 4 °C to allow sEV precipitation.

The sEV-containing pellet was harvested by centrifugation at 10,000× *g* for 1 h at 4 °C, washed twice with sterile phosphate-buffered saline (PBS) (Solarbio, Beijing, China) via repeated centrifugation under the same conditions, and finally resuspended in PBS for subsequent experiments. Notably, negative markers of non-vesicular contaminants were not evaluated in this study; therefore, co-isolation of non-vesicular components cannot be excluded. The isolation procedure was repeated in at least three independent biological experiments using different cell passages.

### 2.4. Transmission Electron Microscopy (TEM)

The morphology of sEVs was characterized by transmission electron microscopy (TEM). The sEV suspension was diluted to an appropriate concentration with PBS. A 20 µL aliquot was placed onto a 200-mesh copper grid and allowed to adsorb for 1 min at room temperature. Excess liquid was gently absorbed away with filter paper. The grid was then negatively stained with 2% phosphotungstic acid (pH 7.0) for 5 min at room temperature. After removing residual staining solution and air-drying for 2 min, the grid was observed and photographed under a Tecnai G2 Spirit TWIN transmission electron microscope (FEI Company, Hillsboro, OR, USA) at an accelerating voltage of 80 kV. The testing was performed with at least three independent biological experiments, with three technical replicates each.

### 2.5. Dynamic Light Scattering (DLS)

The hydrodynamic size distribution of sEVs was characterized by dynamic light scattering (DLS) using a Zetasizer Nano (Malvern Panalytical, Malvern, Worcestershire, UK). Briefly, the sEV suspension was diluted with particle-free PBS to an optimal concentration for single-particle detection. An aliquot of the diluted sample was loaded into the measurement cuvette. The intensity fluctuations of scattered light were recorded. The hydrodynamic diameter and particle concentration were subsequently calculated by the integrated software based on the Stokes–Einstein equation. The testing was performed with at least three independent biological experiments, with three technical replicates each.

### 2.6. Western Blotting

Total protein was extracted from cells or sEVs. Briefly, samples were lysed on ice with RIPA lysis buffer (Beyotime, Shanghai, China) supplemented with protease inhibitors, and the lysates were clarified by centrifugation (12,000× *g*, 15 min, 4 °C). The protein content in each supernatant was determined with a BCA protein assay kit (Beyotime, Shanghai, China).

Equal amounts of protein (20–30 µg) were mixed with 5 × SDS loading buffer, denatured at 95 °C for 10 min, and separated by SDS-PAGE on 8–15% gels. The resolved proteins were then electrotransferred onto polyvinylidene difluoride (PVDF) membranes (Millipore, Burlington, MA, USA). To block nonspecific binding, the membranes were soaked in 5% (*w*/*v*) skim milk for 1 h at room temperature, followed by overnight incubation at 4 °C with the following primary antibodies diluted in blocking solution: anti-CD81 (1:1000; Boster, Wuhan, China), anti-TSG101 (1:1000; Boster, Wuhan, China), anti-CD63 (1:1000; Boster, Wuhan, China), anti-LC3B (1:1000; Abmart, Shanghai, China), anti-p62 (1:6000; Abmart, Shanghai, China), and anti-β-ACTIN (1:1000; Beyotime, Shanghai, China).

After incubation with horseradish peroxidase (HRP)-conjugated secondary antibodies (Beyotime, Shanghai, China), the immunoreactive bands were visualized using enhanced chemiluminescence (ECL) (Beyotime, Shanghai, China) with a gel documentation system. All immunoblotting experiments were repeated at least three times using independently prepared cell lysates (biological replicates), and representative blots are shown in the figures. Band density quantification was performed using ImageJ software (version 1.54t).

### 2.7. H_2_O_2_-Induced Oxidative Injury Model and Experimental Grouping

To establish an oxidative damage model of SH-SY5Y cells, H_2_O_2_ was freshly diluted in serum-free α-MEM. SH-SY5Y cells were treated with H_2_O_2_ at gradient concentrations (200, 400, 800, and 1600 μM) for 2 h at 37 °C. Cells cultured in serum-free α-MEM without H_2_O_2_ served as the control group. After H_2_O_2_ stimulation, the medium was discarded, cells were washed twice with sterile PBS, and fresh complete medium was added for subsequent experiments. Functional assays were performed to verify successful establishment of the oxidative injury model.

To evaluate the protective effect of DPSC-sEVs against H_2_O_2_-induced oxidative damage in SH-SY5Y cells, cells were pre-incubated with DPSC-sEVs at gradient concentrations (6.25, 12.5, 25, 50, 100, 200, and 300 μg/mLmL) for 24 h, followed by stimulation with 400 μM H_2_O_2_ for 2 h. The medium was then replaced with fresh complete medium, and cells were cultured for another 24 h before subsequent detection. SH-SY5Y cells treated with 400 μM H_2_O_2_ alone (without sEV preconditioning) served as the injury control.

To further verify that sEV-carried miR-100-5p mediated the neuroprotective effect against H_2_O_2_-induced oxidative damage, the following experimental groups were set up: normal culture group; H_2_O_2_ injury group; H_2_O_2_ + sEV group; H_2_O_2_ + sEV + miR-100-5p inhibitor group; H_2_O_2_ + inhibitor negative control (inhibitor-NC) group. After transfection, cells were pretreated with DPSC-sEVs (where indicated) and then exposed to H_2_O_2_ (except the normal culture group) for oxidative injury induction, followed by subsequent functional and molecular detection.

All subsequent cellular functional detection and molecular index detection were performed strictly following the above grouping design. All experiments were performed in three independent biological replicates, with five technical replicates per group.

### 2.8. Cell Viability Assay

Cell viability was assessed using the Cell Counting Kit-8 (CCK-8) method. Following the indicated treatments, 10 µL of CCK-8 reagent (Beyotime, Shanghai, China) was added to each well of SH-SY5Y cells cultured in 96-well plates. After incubation at 37 °C for 2 h, the absorbance at 450 nm was measured using a Varioskan Flash microplate reader (Thermo Scientific, Vantaa, Finland). Cell viability was normalized to the control group. Each experiment included five technical replicates per group, and all results were verified in at least three independent biological experiments using different cell passages.

### 2.9. Intracellular ROS Detection

Intracellular ROS levels were measured using the fluorescent probe 2′, 7′-dichlorodihydrofluorescein diacetate (DCFH-DA) (Beyotime, Shanghai, China). Briefly, SH-SY5Y cells were seeded in 6-well plates and subjected to the indicated treatments. Cells were incubated with 10 µM DCFH-DA (diluted 1:1000 in serum-free medium) at 37 °C for 30 min in the dark. After incubation, cells were rinsed three times with PBS to remove unbound probe. For quantitative analysis, cells were trypsinized, resuspended in PBS, and immediately analyzed on a flow cytometer (BD Accuri C6, BD Biosciences, Franklin Lakes, NJ, USA), with excitation and emission wavelengths set at 488 nm and 525 nm, respectively. For qualitative visualization, fluorescent images were captured using a fluorescence microscope (SOPTOP, Sunny Optical Instrument Co., Ltd., Yuyao, China) under the same filter set. Each experiment included three technical replicates per group, and all results were verified in at least three independent biological experiments using different cell passages.

### 2.10. LDH Cytotoxicity Assay

Cytotoxicity was evaluated by quantifying lactate dehydrogenase (LDH) release into the culture medium with a commercial LDH assay kit (Beyotime, Shanghai, China), following the manufacturer’s protocol. Briefly, after the indicated treatments, the culture supernatant was collected from each well of a 96-well plate. The supernatant was incubated with the reaction mixture for 30 min at room temperature in the dark. Absorbance was measured at 490 nm (with a reference wavelength of 600 nm to subtract background) using a microplate reader. Cytotoxicity percentage was calculated using the following formula: Cytotoxicity (%) = [(Absorbance of experimental group − Absorbance of spontaneous LDH release control)/(Absorbance of maximum LDH release control − Absorbance of spontaneous LDH release control)] × 100%. Each experiment included five technical replicates per group, and all results were verified in at least three independent biological experiments using different cell passages.

### 2.11. Apoptosis Assay

Cell apoptosis was quantified by flow cytometry using an Annexin V-FITC/PI apoptosis detection kit (Beyotime, Shanghai, China). Briefly, SH-SY5Y cells were seeded in 24-well plates and subjected to the indicated treatments. After treatment, both adherent and floating cells were harvested, washed twice with cold PBS, and resuspended in 195 µL of 1× Annexin V binding buffer. Subsequently, 5 µL of Annexin V-FITC and 10 µL of propidium iodide (PI) staining solution were added to the cell suspension. After gentle mixing, cells were incubated for 15 min at ambient temperature in the dark. Apoptosis analysis was performed within 1 h using a BD Accuri C6 flow cytometer (BD Biosciences, USA). Data were analyzed with FlowJo software v10.7 (FlowJo, LLC, Ashland, OR, USA), and cells were classified into four populations: viable cells (Annexin V^−^/PI^−^), early apoptotic cells (Annexin V^+^/PI^−^), late apoptotic/necrotic cells (Annexin V^+^/PI^+^), and dead cells (Annexin V^−^/PI^+^, if present). Each experiment included three technical replicates per group, and all results were verified in at least three independent biological experiments using different cell passages.

### 2.12. Malondialdehyde (MDA) Assay

Lipid peroxidation level was assessed by measuring malondialdehyde (MDA) content using a commercial assay kit (Solarbio, Beijing, China), based on the thiobarbituric acid (TBA) method. Briefly, after the indicated treatments, cells were collected and lysed. The cell lysates were then centrifuged at 12,000× *g* for 10 min at 4°C to obtain clear supernatants. Following the kit manufacturer’s instructions, the supernatant was mixed with TBA reagent in a test tube and heated in a 95°C water bath for 60 min to allow formation of the MDA-TBA adduct. After the mixture was cooled to room temperature, absorbance at 532 nm was measured using a microplate reader. MDA concentration was normalized to the total protein content of each sample and expressed as nmol per milligram of protein (nmol/mg protein). Each experiment included five technical replicates per group, and all results were verified in at least three independent biological experiments using different cell passages.

### 2.13. Antioxidant Enzyme Activity Assays

The activities of two key antioxidant enzymes, superoxide dismutase (SOD) and glutathione peroxidase (GSH-Px), were measured using commercial assay kits. After treatment, SH-SY5Y cells were harvested, washed with cold PBS, and lysed by ultrasonication on ice. The homogenates were centrifuged at 12,000× *g* for 15 min at 4°C. The resulting supernatants were collected for enzymatic activity assays. Total SOD activity was determined using the Total Superoxide Dismutase Assay Kit (hydroxylamine method; Nanjing Jiancheng Bioengineering Institute, Nanjing, China), which measures the ability of SOD to inhibit superoxide anion-induced oxidation of hydroxylamine. GSH-Px activity was assessed using the Glutathione Peroxidase Assay Kit (colorimetric method; Nanjing Jiancheng Bioengineering Institute, Nanjing, China), which quantifies the oxidation rate of reduced glutathione (GSH) to oxidized glutathione (GSSG) coupled with H_2_O_2_ decomposition. All enzymatic activities were measured according to the manufacturers’ protocols. The results were normalized to the total protein concentration of each sample (determined by BCA assay) and expressed as units per milligram of protein (U/mg protein). Each experiment included five technical replicates per group, and all results were verified in at least three independent biological experiments using different cell passages.

### 2.14. Quantitative Real-Time PCR (qPCR) for miRNA Expression Analysis

Total RNA was extracted from cells or sEVs using AG RNAex Pro reagent according to the manufacturer’s protocol. RNA concentration and purity were assessed using a Nano-300 spectrophotometer (Aosheng, Hangzhou, China); samples with an A260/A280 ratio ranging from 1.8 to 2.0 were considered acceptable for subsequent analysis. For miRNA-specific reverse transcription, 1 µg of total RNA was reverse-transcribed into cDNA using the miRNA cDNA First Strand Synthesis Kit (AG11745; Accurate Biology, Changsha, China) with stem-loop RT primers. Quantitative real-time PCR (qPCR) was subsequently performed using the SYBR Green Pro Taq HS Premix qPCR Kit II (AG11701; Accurate Biology, Changsha, China). The 20 µL reaction mixture consisted of 10 µL of 2× SYBR Green Premix, 0.8 µL of forward and reverse primers (10 µM each), 2 µL of cDNA template, and 6.4 µL of RNase-free water. The thermal cycling conditions were as follows: initial denaturation at 95 °C for 30 s; followed by 40 cycles of 95 °C for 5 s and 60 °C for 30 s. Melting curve analysis was performed at the end of each run to confirm amplification specificity. The small nuclear RNA U6 was used as an endogenous reference for normalization. Relative expression levels of target miRNAs were calculated using the 2^(−ΔΔCt)^ method. The specific primer sequences are shown in Table 1. Each sample was analyzed in three technical replicates, and all experiments were independently repeated at least three times with separate RNA preparations.

### 2.15. Dual-Luciferase Reporter Assay

To verify the direct targeting of the mTOR 3′ untranslated region (3′UTR) by miR-100-5p t, a dual-luciferase reporter assay was performed. Wild-type (h-mTOR-3′UTR-wt) and mutant (h-mTOR-3′UTR-mut) fragments of the human mTOR 3′UTR were synthesized and inserted into the pmirGLO dual-luciferase vector (Promega, Madison, USA) of Qingke Biotechnology Co., Ltd. (Beijing, China). HEK-293T cells were seeded in 96-well plates. Twenty-four hours later, cells were co-transfected with the reporter plasmids (wt or mut) and either the miR-100-5p mimic or mimic negative control using Lipofectamine 2000, according to the manufacturer’s instructions. At 48 h post-transfection, cells were lysed, and firefly and Renilla luciferase activities were measured sequentially using the Dual Luciferase Reporter Assay Kit (Heruibio, Fuzhou, China) with a Varioskan Flash microplate reader (Thermo Scientific, Vantaa, Finland). Firefly luciferase activity was normalized to Renilla luciferase activity (internal control) for each well. Relative luciferase activity was expressed as the ratio of the normalized value in the mimic group to that in the negative control group. Each transfection condition was set up in triplicate technical replicates, and the assay was independently repeated at least three times with independently prepared plasmid and cell batches.

### 2.16. Transfection of miRNA-100-5p Mimics and Inhibitors

For mechanistic rescue experiments, SH-SY5Y cells were subjected to liposomal transfection to overexpress or silence miR-100-5p. Four transfection subgroups were set as follows: (1) mimic-NC group: cells transfected with negative control mimics; (2) miR-100-5p mimic group: cells transfected with miR-100-5p mimics to upregulate intracellular miR-100-5p levels; (3) inhibitor-NC group: cells transfected with negative control inhibitor; (4) miR-100-5p inhibitor group: cells transfected with miR-100-5p inhibitor to knock down endogenous miR-100-5p.

SH-SY5Y cells were transfected at 70% confluence using Lipofectamine 2000 (Thermo Fisher Scientific, Waltham, MA, USA) according to the manufacturer’s guidelines. Briefly, 30 nM of miR-100-5p mimic, inhibitor, or their scrambled negative controls were pre-complexed with the transfection reagent in Opti-MEM medium, then added to the cells. After 6 h of incubation, the transfection medium was replaced with fresh complete medium. Cells were harvested for analysis at 24 h post-transfection. All oligonucleotides were obtained from AG Biotech Co., Ltd. (Changsha, China). The sequences of synthetic miRNA oligonucleotides are shown in Table 2. Each transfection condition was set up in triplicate technical replicates, and the assay was repeated independently at least three times with independently prepared cell batches.

### 2.17. Monodansylcadaverine (MDC) Staining

Autophagic activity was evaluated by detecting autophagic vacuole accumulation using MDC staining. SH-SY5Y cells were seeded on coverslips in 24-well plates and subjected to the indicated treatments. After treatment, cells were incubated with 50 µM MDC (MedChemExpress, Shanghai, China) in serum-free medium for 30 min at 37 °C in the dark. Subsequently, cells were washed twice with pre-warmed PBS, fixed with 4% paraformaldehyde (PFA) for 15 min at room temperature, and washed three times with PBS. Fluorescent images were captured immediately using a fluorescence microscope (ZEISS, Oberkochen, Germany) with a standard DAPI filter set (excitation: 335–380 nm; emission: 420–470 nm). The number and intensity of MDC-positive puncta in the cytoplasm were analyzed using ImageJ software.

### 2.18. Statistical Analysis

All data are presented as mean ± standard deviation (SD), derived from at least three independent experiments. Technical replicates from a single experiment were averaged prior to statistical analysis across biological replicates. All statistical analyses were performed using GraphPad Prism 10.0. Differences between two groups were evaluated by Student’s two-tailed unpaired *t*-test. Differences among multiple groups were analyzed by one-way ANOVA followed by Tukey’s multiple comparisons post hoc test. No significant difference is marked as “ns”. A *p*-value less than 0.05 was considered statistically significant. Significance levels are denoted as follows: * *p* < 0.05, ** *p* < 0.01, *** *p* < 0.001, **** *p* < 0.0001.

## 3. Results

### 3.1. Characterization of DPSCs

**Figure 1:**
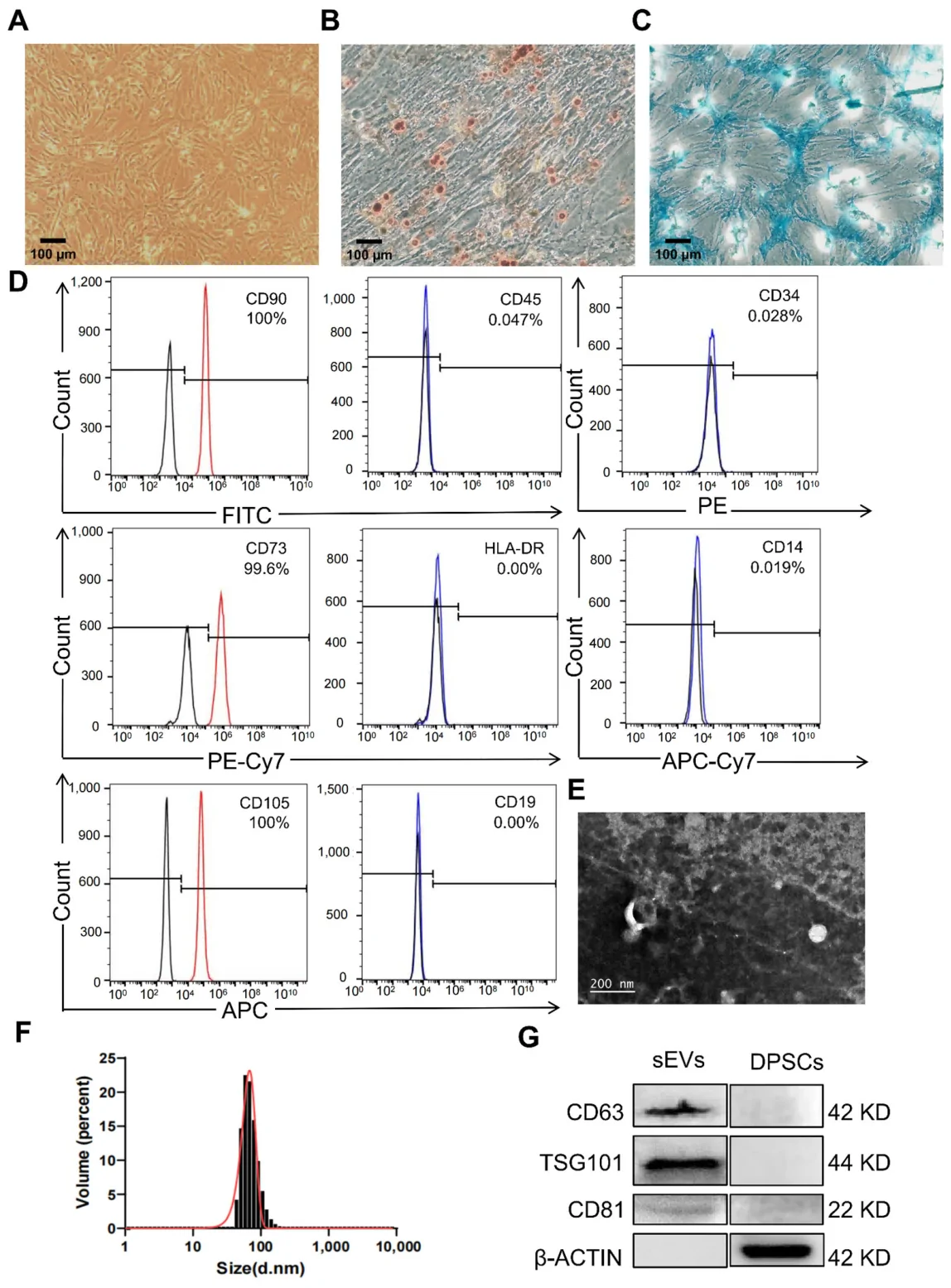
Isolation and characterization of dental pulp stem cells (DPSCs) and DPSC-derived small extracellular vesicles (DPSC-sEVs). (**A**) Representative bright-field micrograph showing the morphology of cultured DPSCs (scale bar = 100 μm). (**B**) Osteogenic differentiation capacity of DPSCs after 21 days of induction, detected by Alizarin Red S staining for calcium nodule formation (red) (scale bar = 100 μm). (**C**) Chondrogenic differentiation of DPSCs after 21 days of induction, evaluated by Alcian Blue staining for cartilage-specific glycosaminoglycans (blue) (scale bar = 100 μm). (**D**) Flow cytometry analysis of surface marker expression in DPSCs. Cells were positive for mesenchymal stem cell markers CD73, CD90 and CD105 and negative for hematopoietic markers CD14, CD19, CD34, and CD45 as well as the immune marker HLA-DR. (**E**) Transmission electron microscopy (TEM) image of DPSC-sEVs, displaying the typical cup-shaped morphology of extracellular vesicles (scale bar = 200 nm). (**F**) Hydrodynamic size distribution of sEVs determined by dynamic light scattering (DLS). The plot shows volume-weighted size distribution of sEVs. (**G**) Western blot analysis of canonical sEV protein markers (CD63, CD81, TSG101) in DPSC-sEVs and whole cell lysates, with β-ACTIN as the loading control. All experiments were performed with three independent biological replicates, and three technical replicate wells were included for each sample in each assay.

DPSCs adhered to culture plastic within 4–6 h after seeding and reached complete attachment by 12 h. After 24 h of culture, cells were fully spread and displayed a typical fibroblast-like spindle-shaped morphology under standard culture conditions, with a centrally located oval nucleus and elongated cytoplasmic processes (Figure 1A). Cells reached 80–90% confluence at approximately 72 h and were ready for subculture.

To assess multipotent differentiation potential, DPSCs were subjected to osteogenic and chondrogenic induction. After 3 weeks of induction, osteogenic cultures formed distinct mineralized nodules that stained positive with Alizarin Red S (Figure 1B), while chondrogenic cultures exhibited prominent deposition of glycosaminoglycans, as verified by Alcian Blue staining (Figure 1C). No positive staining was observed in non-induced control cultures, confirming the differentiation was induction-dependent.

Flow cytometric immunophenotyping confirmed the mesenchymal stem cell identity of the isolated cells. The cells were strongly positive (>95%) for the canonical mesenchymal markers CD73, CD90, and CD105. Conversely, they were negative (<2%) for hematopoietic lineage markers (CD14, CD19, CD34, CD45) and the major histocompatibility complex class II molecule HLA-DR (Figure 1D).

Collectively, these characteristics—plastic adherent growth, fibroblast-like spindle morphology, osteogenic and chondrogenic differentiation capacity, and characteristic immunophenotype—confirm the successful isolation and characterization of human DPSCs, which were subsequently used for sEV isolation.

### 3.2. Isolation and Characterization of DPSC-sEVs

The isolated particles were identified as sEVs based on comprehensive morphological, biophysical, and biochemical characterization. TEM revealed that the particles exhibited the typical cup-shaped or saucer-like morphology of sEVs, with diameters ranging from 30 to 200 nm (Figure 1E). DLS further quantified the size distribution, showing a predominant peak at approximately 100 nm (Figure 1F), which is consistent with the established size range for sEVs. Western blot analysis confirmed the enrichment of sEV-specific protein markers. As shown in Figure 1G, the sEV fraction showed strong positive signals for the transmembrane proteins CD63 and CD81 as well as the cytosolic protein TSG101; in contrast, these markers were only weakly detected in whole-cell lysates of DPSCs. Taken together, these morphological, size, and marker profiling data verify the successful isolation of sEVs from DPSC culture supernatants.

### 3.3. Establishment and Validation of an H_2_O_2_-Induced Oxidative Stress Model in SH-SY5Y Cells

**Figure 2:**
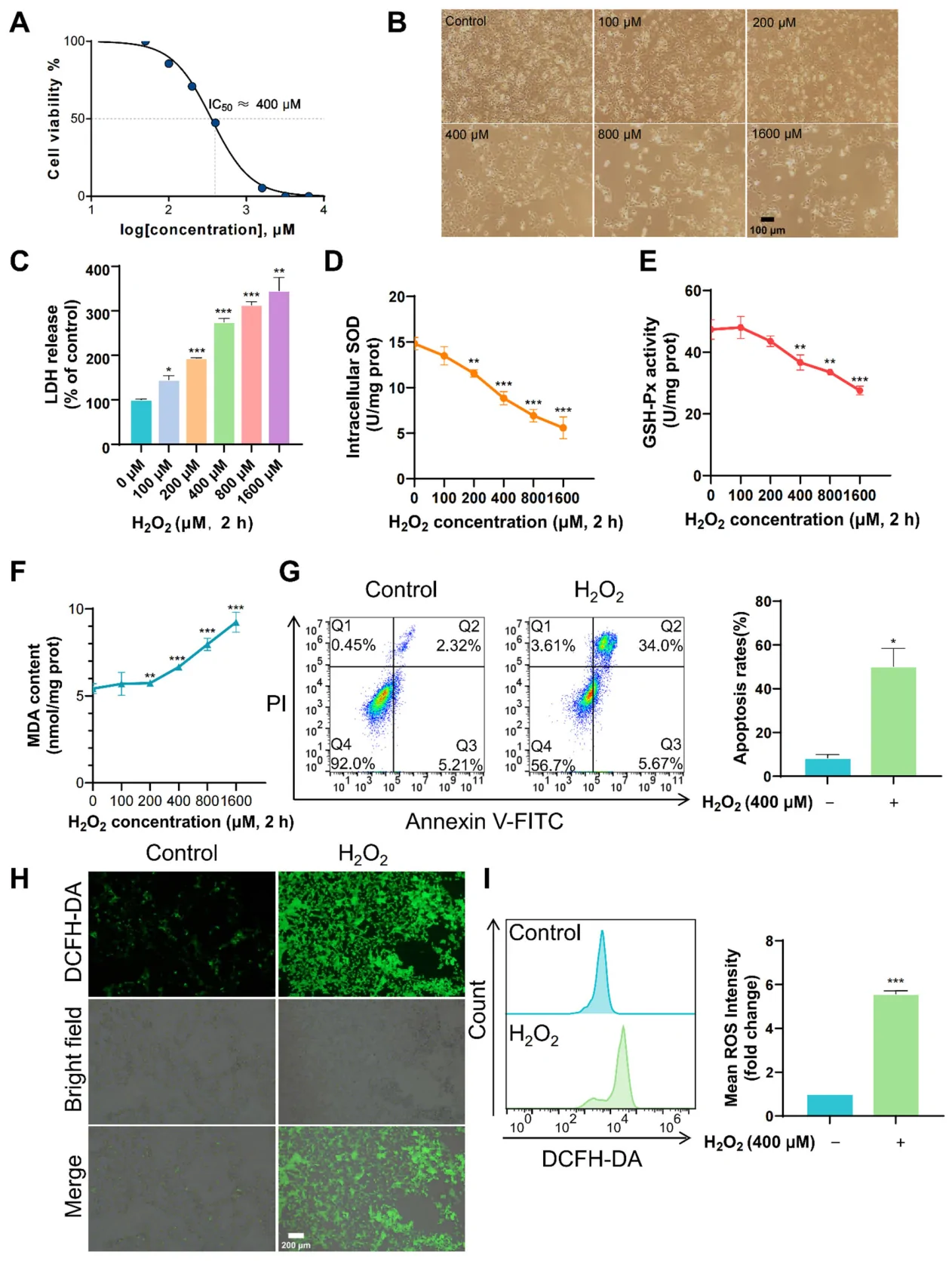
Establishment and characterization of an H_2_O_2_-induced oxidative stress injury model in SH-SY5Y cells. (**A**) Cell viability of SH-SY5Y cells after 24 h exposure to gradient concentrations of H_2_O_2_, determined by CCK-8 assay. Cell viability decreased in a concentration-dependent manner, with a half-maximal inhibitory concentration (IC_50_) calculated as approximately 400 µM. (**B**) Bright-field micrographs showing morphological changes and density alterations of SH-SY5Y cells after 24 h exposure to gradient concentrations of H_2_O_2_: 0 µM (control), 100 µM, 200 µM, 400 µM, 800 µM and 1600 µM, respectively (scale bar = 100 μm). (**C**) Quantitative analysis of lactate dehydrogenase (LDH) release in cell culture supernatant following 24 h H_2_O_2_ exposure. Intracellular antioxidant enzyme activities in cell lysates: (**D**) superoxide dismutase (SOD) activity and (**E**) glutathione peroxidase (GSH-Px) activity. (**F**) Quantification of intracellular malondialdehyde (MDA) level, a well-established marker of lipid peroxidation. (**G**) Total apoptosis rate of SH-SY5Y cells analyzed by flow cytometry with Annexin V-FITC/PI double staining. Dot-plot color gradient: Blue→green→yellow→red represents increasing cell density. (**H**) Representative fluorescence micrographs of intracellular reactive oxygen species (ROS) stained with DCFH-DA staining (green) (scale bar = 200 μm). (**I**) Quantitative analysis of mean intracellular ROS fluorescence intensity by flow cytometry with DCFH-DA staining. Data are presented as the mean ± SD. *n* = 3 represents independent biological replicates using different passages of SH-SY5Y cells, with five technical replicate wells per sample (**A**–**F**) or three technical replicate wells per sample (**G**,**H**) in each experiment. Differences between two groups were analyzed by Student’s *t*-test (**G**–**I**), and differences across multiple groups were analyzed by one-way ANOVA followed by Tukey’s multiple comparisons post hoc test (**A**–**F**). * *p* < 0.05; ** *p* < 0.01; *** *p* < 0.001.

To establish a neuronal oxidative damage model, SH-SY5Y cells were treated with escalating concentrations of H_2_O_2_ for 2 h; H_2_O_2_ is a representative reactive oxygen species whose overproduction is implicated in neurodegenerative pathologies. Cell viability assays revealed a dose-dependent decrease in survival following treatment with 100 to 1600 µM H_2_O_2_ (Figure 2A). A concentration of 400 µM H_2_O_2_, which reduced cell viability to approximately 50% (i.e., the half-maximal inhibitory concentration, IC_50_), was selected for all subsequent experiments to induce sublethal oxidative injury.

Microscopic examination confirmed the cytotoxic effects, showing a progressive reduction in cell density and a shrunken, aggregated morphology with increasing H_2_O_2_ concentration (Figure 2B). This morphological damage was corroborated by a significant increase in LDH release, indicating compromised membrane integrity (Figure 2C). Comprehensive biochemical analysis further verified the successful induction of oxidative stress. Treatment with 400 µM H_2_O_2_ triggered a substantial elevation in intracellular ROS levels (Figure 2H,I) and the lipid peroxidation product MDA (Figure 2F). Concurrently, the activities of key antioxidant enzymes SOD and GSH-Px were markedly diminished (Figure 2D,E). This redox imbalance culminated in a significant increase in the apoptosis rate (Figure 2G). Collectively, these data confirm the successful establishment of a reliable oxidative stress model in SH-SY5Y cells using 400 µM H_2_O_2_.

### 3.4. DPSC-sEVs Attenuate H_2_O_2_-Induced Cytotoxicity and Apoptosis

**Figure 3:**
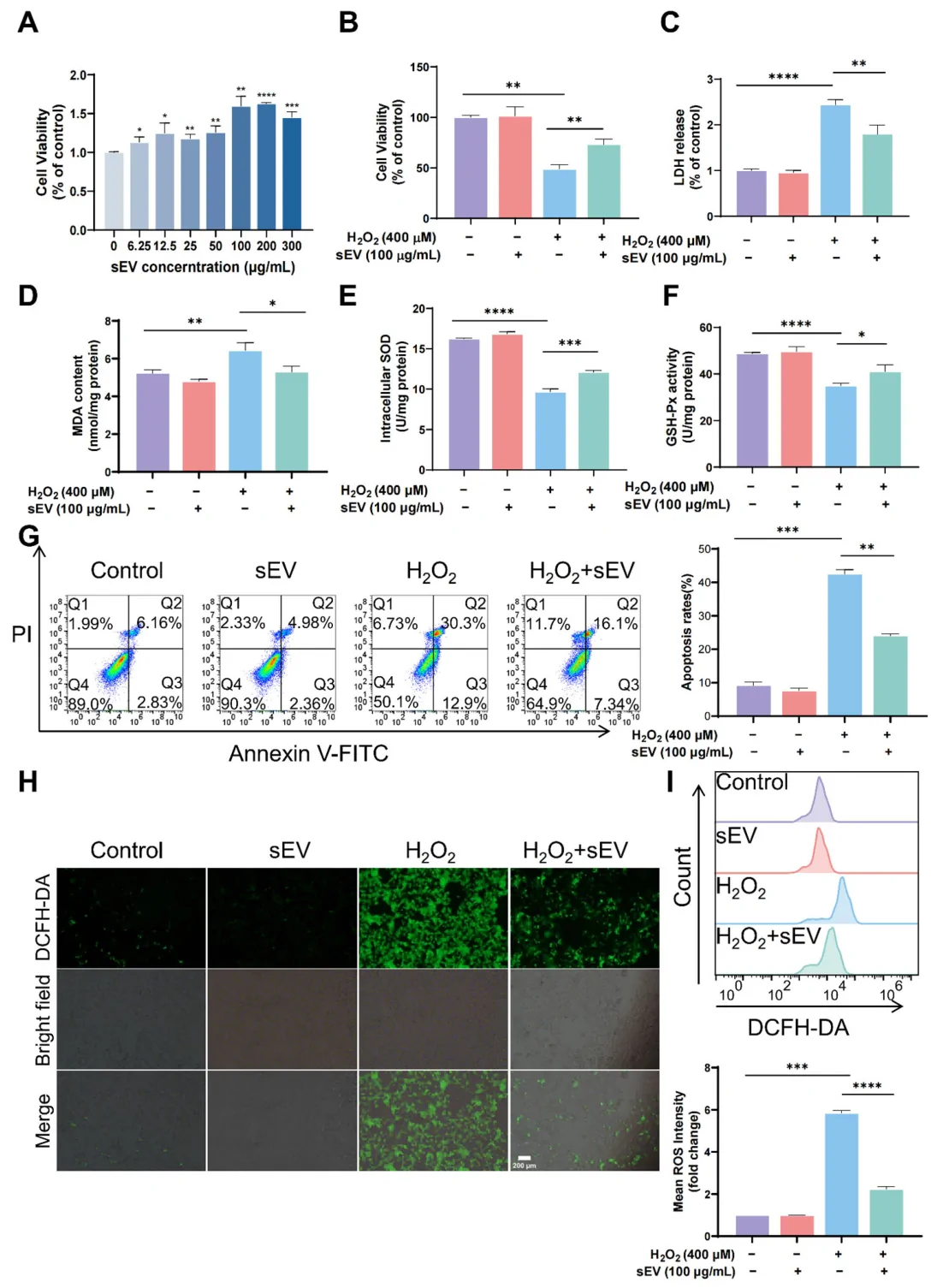
DPSC-derived small extracellular vesicles (DPSC-sEVs) exert protective effects against H_2_O_2_-induced oxidative damage in SH-SY5Y cells. (**A**) Cell viability of SH-SY5Y cells determined by CCK-8 assay. Cells were pre-incubated with gradient concentrations of DPSC-sEVs for 24 h, followed by 2 h of oxidative challenge with 400 µM H_2_O_2_. (**B**) Cell viability following pretreatment with the optimized concentration of 100 µg/mL DPSC-sEVs and subsequent 2 h exposure to 400 µM H_2_O_2_. (**C**) Quantitative detection of lactate dehydrogenase (LDH) release in cell culture supernatant, an indicator of plasma membrane damage. (**D**) Intracellular malondialdehyde (MDA) content, a marker of lipid peroxidation. Endogenous antioxidant enzyme activities in SH-SY5Y cell lysates: (**E**) superoxide dismutase (SOD) and (**F**) glutathione peroxidase (GSH-Px). (**G**) Apoptosis rate analyzed by flow cytometry with Annexin V-FITC/PI staining. Dot-plot color gradient: Blue→green→yellow→red represents increasing cell density. (**H**) Representative fluorescence micrographs of intracellular reactive oxygen species (ROS) stained with DCFH-DA (green) (scale bar = 200 μm). (**I**) Quantitative analysis of intracellular ROS levels by flow cytometry with DCFH-DA staining. Data are presented as the mean ± SD. *n* = 3 refers to three independent biological replicates (separate experimental runs using different batches of SH-SY5Y cells and DPSC-sEV preparations), with five technical replicate wells per sample (**A**–**F**) or three technical replicate wells per sample (**G**,**H**) in each experiment. Differences were analyzed by one-way ANOVA followed by Tukey’s post hoc test. * *p* < 0.05; ** *p* < 0.01; *** *p* < 0.001; **** *p* < 0.0001.

Pretreatment with DPSC-sEVs (6.25–300 µg/mL) for 24 h significantly improved the viability of SH-SY5Y cells subsequently exposed to 400 µM H_2_O_2_ (Figure 3A). A concentration of 100 µg/mL DPSC-sEVs was selected for subsequent experiments based on its robust protective efficacy. Compared to cells treated with H_2_O_2_ alone, pretreatment with DPSC-sEVs markedly increased cell survival (Figure 3B) and concurrently decreased the apoptotic rate (Figure 3G), demonstrating a protective effect against oxidative stress-induced cell death.

### 3.5. DPSC-sEVs Mitigate Oxidative Stress and Membrane Damage

DPSC-sEV pretreatment effectively counteracted the redox imbalance induced by H_2_O_2_. The H_2_O_2_-triggered surge in intracellular ROS was significantly suppressed by DPSC-sEVs (Figure 3H,I). This reduction in oxidative burden was further supported by decreased lipid peroxidation, as evidenced by lower MDA levels in the DPSC-sEV-pretreated group compared with the H_2_O_2_-only group (Figure 3D). Additionally, DPSC-sEV pretreatment preserved cell membrane integrity, as indicated by a significant reduction in LDH release following H_2_O_2_ challenge (Figure 3C).

### 3.6. DPSC-sEVs Restore Antioxidant Enzyme Activities

The activities of key antioxidant enzymes were impaired by H_2_O_2_ but partially restored by DPSC-sEVs. Treatment with H_2_O_2_ alone significantly inhibited the activities of SOD and GSH-Px. In contrast, cells pretreated with DPSC-sEVs prior to H_2_O_2_ exposure maintained significantly higher activities of both enzymes (Figure 3E,F), indicating that DPSC-sEVs bolster the endogenous cellular antioxidant defense system.

### 3.7. DPSC-sEVs Enhance Autophagic Activity in Oxidatively Stressed SH-SY5Y Cells

Given that previous studies have documented a close association between sEV-mediated antioxidant effects and autophagy regulation, we next investigated whether these sEVs modulate autophagy in H_2_O_2_-exposed SH-SY5Y cells. To this end, we assessed the expression of key autophagy markers.

**Figure 4:**
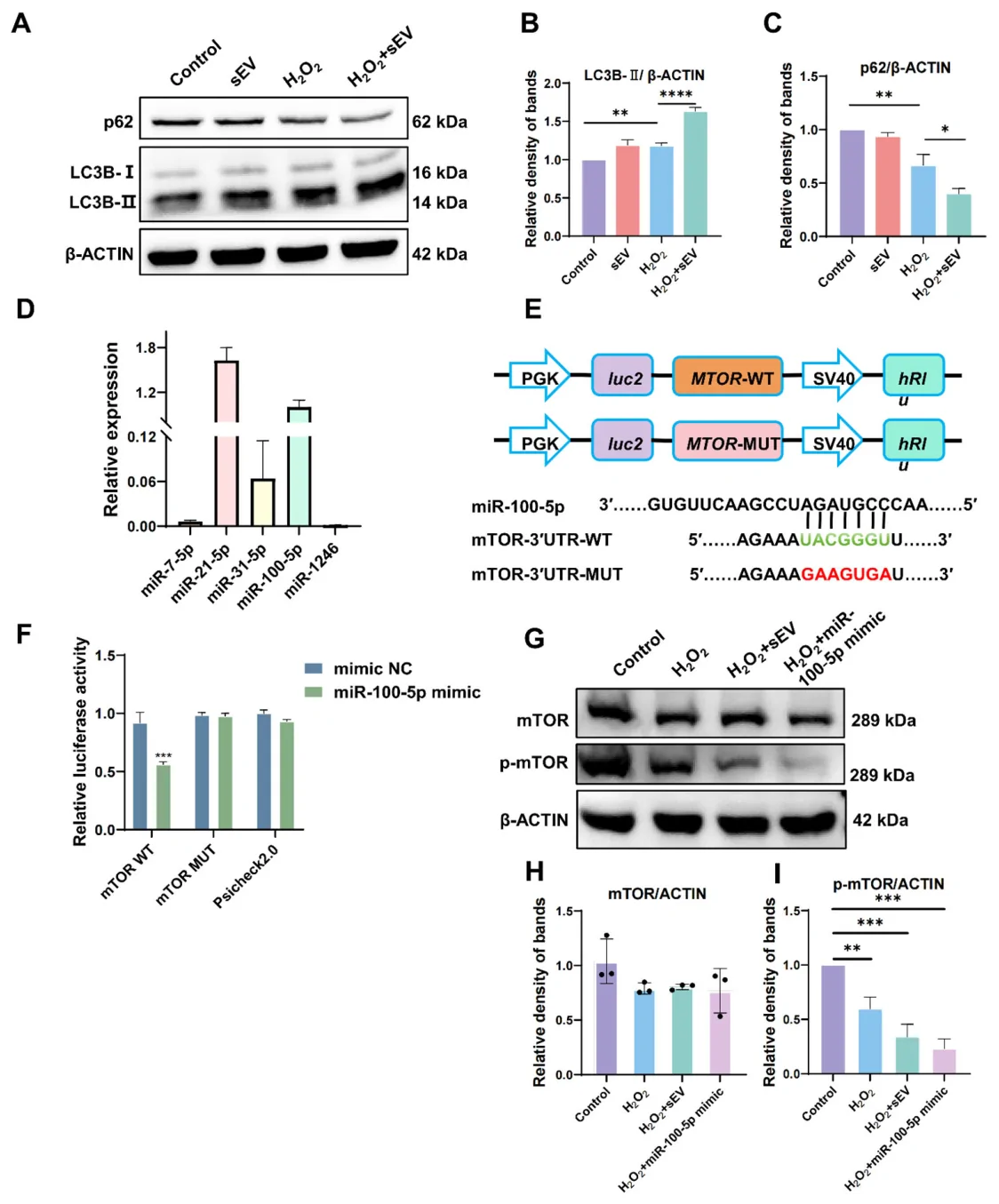
miR-100-5p mediates DPSC-sEV-enhanced autophagy in SH-SY5Y cells under oxidative stress by targeting the human *MTOR* gene. (**A**–**C**) Western blot analysis of autophagy-related proteins LC3B and p62 in SH-SY5Y cells. Cells were pretreated with or without 100 µg/mL DPSC-sEVs for 24 h, followed by 2 h exposure to 400 µM H_2_O_2_. β-ACTIN served as the internal loading control. (**D**) Relative expression of candidate miRNAs in DPSC-sEVs, detected by quantitative real-time PCR (qPCR). (**E**) Schematic diagram showing the predicted wild-type (WT) and mutant (Mut) miR-100-5p binding sites in the 3′ untranslated region (3′UTR) of the human *MTOR* gene. (**F**) Dual-luciferase reporter assay verifying direct targeting of the *MTOR* 3′UTR by miR-100-5p. HEK-293T cells were co-transfected with the miR-100-5p mimic (or mimic negative control, NC) and a reporter plasmid containing either the WT or Mut *MTOR* 3′UTR sequence. Firefly luciferase activity was normalized to Renilla luciferase activity. (**G**) Western blot analysis of mTOR and phosphorylated mTOR (p-mTOR) expression in SH-SY5Y cells under the indicated treatments. All quantitative data are presented as the mean ± SD from three independent biological replicates, with triplicate technical replicates for each assay. Differences were analyzed by Student’s *t*-test (**F**), and one-way ANOVA followed by Tukey’s post hoc test (**B**,**C**,**H**,**I**).* *p* < 0.05; ** *p* < 0.01; *** *p* < 0.001; **** *p* < 0.0001. DPSC-sEV: dental pulp stem cell-derived small extracellular vesicles; HEK-293T, human embryonic kidney 293T cells; mTOR: mammalian target of rapamycin; Mut, mutant; WT, wild type.

Western blot analysis showed that H_2_O_2_ treatment alone increased the LC3B-II/β-ACTIN ratio and decreased p62 levels compared with the control group, indicating that oxidative stress alone induces autophagy as a cellular stress response. Notably, pretreatment with DPSC-sEVs prior to H_2_O_2_ exposure further elevated the LC3B-II/β-ACTIN ratio and reduced p62 expression to a greater extent than H_2_O_2_ treatment alone (Figure 4A–C). These results indicate that DPSC-sEVs robustly enhance autophagy-related processes in H_2_O_2_-challenged SH-SY5Y cells.

### 3.8. miR-100-5p in DPSC-sEVs Targets the MTOR Gene

We next sought to identify the molecular component within DPSC-sEVs responsible for autophagy activation. Integrative bioinformatic prediction and qPCR validation confirmed that miR-100-5p, miR-21-5p and miR-31-5p were highly enriched in DPSC-sEVs (Table 3, Figure 4D), suggesting their potential roles as functional mediators of sEV biological effects. Consistent with our in vitro functional assays, miR-21-5p and miR-100-5p exerted obvious protective effects against H_2_O_2_-induced oxidative injury in SH-SY5Y neuronal cells, whereas miR-31-5p failed to alleviate neuronal damage under the same oxidative stress conditions (Figure S1). While miR-124-5p has been extensively studied in neuroprotection, with over 2000 publications describing its wide-ranging functions, miR-100-5p remains far less characterized, with fewer than 100 studies to date and particularly limited evidence on neuronal protection. To explore a less-studied, novel regulatory pathway underlying DPSC-sEV-mediated neuroprotection, we selected miR-100-5p as the core research target for subsequent functional and mechanistic experiments.

Bioinformatic prediction using TargetScan and miRanda identified the MTOR—a gene encoding a key negative regulator of autophagy—as a potential target of miR-100-5p (Table 4). This prediction was experimentally verified by a dual-luciferase reporter assay. Co-transfection of the miR-100-5p mimic with a reporter plasmid containing the wild-type MTOR 3′UTR significantly suppressed luciferase activity, whereas no such effect was observed with a plasmid carrying a mutated target site (Figure 4E,F). This confirms that miR-100-5p can directly target the 3′UTR of the MTOR gene.

To further validate the regulatory effect of miR-100-5p on the mTOR pathway, we examined the protein levels of total mTOR and phosphorylated mTOR (p-mTOR) by Western blot (Figure 4G–I). Pretreatment with DPSC-sEVs or transfection with the miR-100-5p mimic induced a moderate downward trend in total mTOR protein levels in SH-SY5Y cells, although the reduction did not reach statistical significance. In contrast, the level of p-mTOR (the functionally active form of mTOR) was significantly decreased by DPSC-sEV pretreatment or with miR-100-5p mimic transfection.

These data suggest that miR-100-5p inactivates the mTOR pathway through both direct translational repression of *MTOR* and indirect modulation of mTOR phosphorylation status. These findings are consistent with the luciferase reporter assay results and confirm that DPSC-sEVs effectively inhibit mTOR phosphorylation in a miR-100-5p-dependent manner, thereby relieving the negative regulation of autophagy.

### 3.9. The Protective Effects of DPSC-sEVs Are Dependent on miR-100-5p

**Figure 5:**
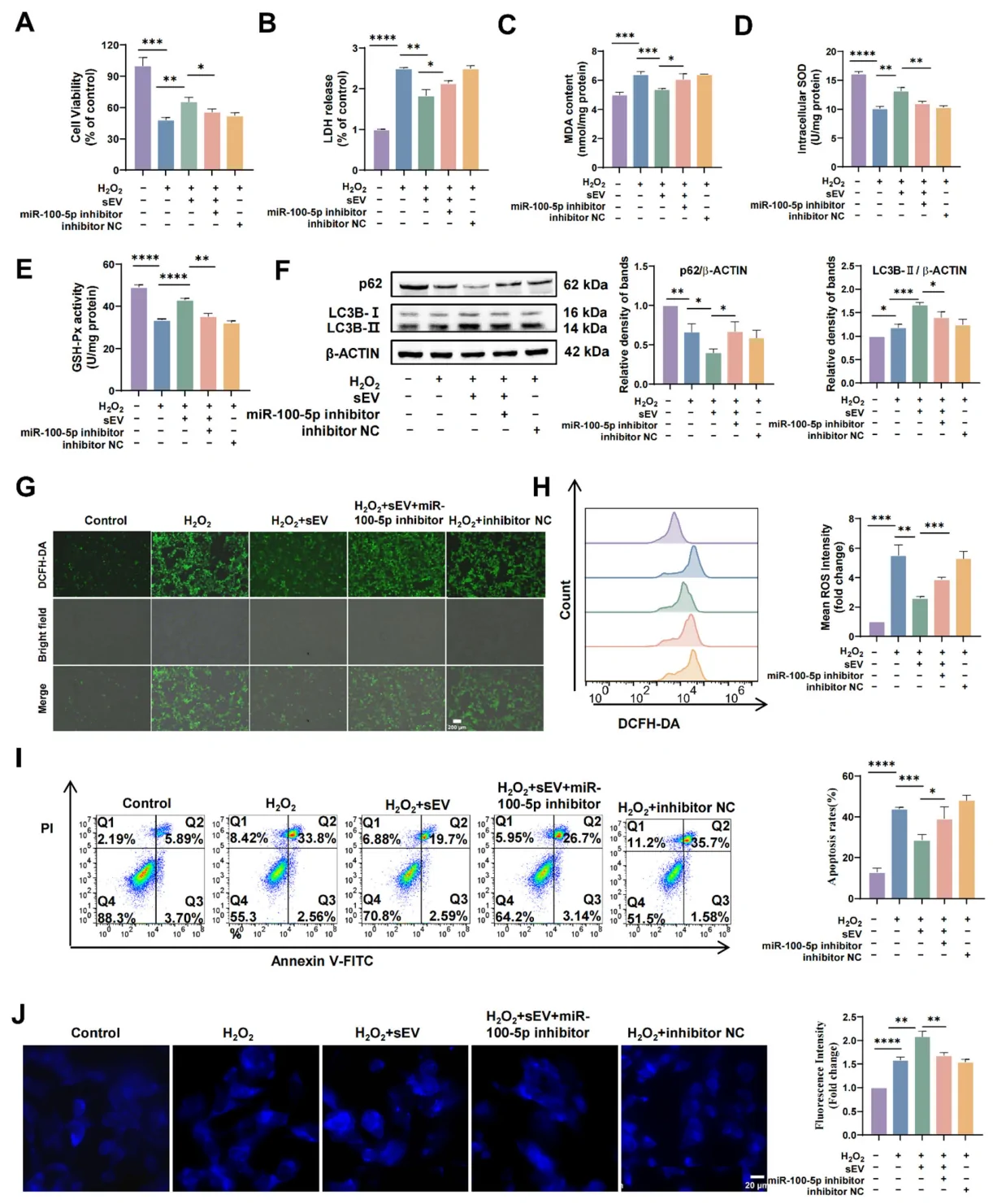
The cytoprotective effect of DPSC-sEVs against H_2_O_2_-induced injury in SH-SY5Y cells is dependent on the carried miR-100-5p. SH-SY5Y cells were transfected with miR-100-5p inhibitor or a matched negative control (inhibitor NC), pretreated with or without 100 µg/mL DPSC-sEVs for 24 h, and then exposed to 400 µM H_2_O_2_ for 2 h to induce oxidative injury. (**A**) Cell viability of each treatment group, determined by CCK-8 assay. (**B**) Lactic dehydrogenase (LDH) release in culture supernatant, an indicator of cell membrane damage. (**C**) Intracellular malondialdehyde (MDA) content, a marker of lipid peroxidation. Activities of the antioxidant enzymes: (**D**) superoxide dismutase (SOD) and (**E**) glutathione peroxidase (GSH-Px). (**F**) Western blot analysis of autophagy-related marker proteins LC3B and p62. β-ACTIN served as the internal loading control. (**G**) Representative fluorescence micrographs of intracellular reactive oxygen species (ROS) stained with DCFH-DA (green) (scale bar = 20 µm). (**H**) Quantitative analysis of intracellular ROS levels by flow cytometry based on DCFH-DA fluorescence intensity. (**I**) Cell apoptosis rate of each group, analyzed by flow cytometry with Annexin V-FITC/PI staining. Dot-plot color gradient: Blue→green→yellow→red represents increasing cell density. (**J**) Representative fluorescence micrographs of intracellular autophagic vacuoles stained with Monodansylcadaverine (MDC) (blue) (scale bar = 20 µm). All quantitative data are presented as the mean ± SD from three independent biological replicates, with five technical replicate wells per sample (**A**–**E**) or three technical replicate wells per sample (**G**–**J**) in each experiment; technical replicates were performed to confirm the band pattern (**F**). Differences were analyzed by one-way ANOVA followed by Tukey’s post hoc test. * *p* < 0.05; ** *p* < 0.01; *** *p* < 0.001; **** *p* < 0.0001. CCK-8, Cell Counting Kit-8; DCFH-DA, 2’, 7’-dichlorodihydrofluorescein diacetate; DPSC-sEV, dental pulp stem cell-derived small extracellular vesicles; NC, negative control.

To determine whether miR-100-5p serves as the critical functional mediator of DPSC-sEVs, we inhibited miR-100-5p in SH-SY5Y cells using a specific inhibitor prior to DPSC-sEV pretreatment and H_2_O_2_ challenge. Pretreatment with DPSC-sEVs significantly attenuated H_2_O_2_-induced cell viability loss, but this protective effect was largely abrogated in cells transfected with the miR-100-5p inhibitor (Figure 5A). Consistently, flow cytometric analysis showed that DPSC-sEVs markedly reduced the H_2_O_2_-induced apoptosis rate, whereas miR-100-5p inhibition reversed this anti-apoptotic effect (Figure 5I). Transfection with the inhibitor negative control (NC) had no such effect, confirming that the reversal of protection was specifically attributable to miR-100-5p knockdown rather than transfection artifacts.

In line with the viability results, the antioxidant effects of DPSC-sEVs were reversed by miR-100-5p inhibition. The DPSC-sEV-mediated reductions in intracellular ROS levels (Figure 5G,H) and lipid peroxidation product MDA (Figure 5C) were significantly attenuated in the inhibitor group. Similarly, the protective effect of DPSC-sEVs on plasma membrane integrity, as indicated by suppressed LDH release, was also compromised following miR-100-5p inhibition (Figure 5B). Furthermore, the DPSC-sEV-induced upregulation of SOD and GSH-Px activities was effectively reversed by the miR-100-5p inhibitor (Figure 5D,E).

Finally, we examined whether miR-100-5p is required for the pro-autophagic activity of DPSC-sEVs. Western blot analysis showed that the DPSC-sEV-induced increase in the LC3B-II/β-actin ratio and decrease in p62 protein level were both significantly counteracted by miR-100-5p inhibition (Figure 5F). This biochemical finding was corroborated by MDC staining: the increase in autophagic puncta induced by DPSC-sEVs was markedly diminished in cells transfected with the miR-100-5p inhibitor (Figure 5J).

### 3.10. miR-100-5p Protects SH-SY5Y Cells from H_2_O_2_-Induced Oxidative Damage via Autophagy Activation

To verify that miR-100-5p alone recapitulates the protective effects of DPSC-sEVs and to investigate the involvement of autophagy in this process, SH-SY5Y cells were transfected with the miR-100-5p mimic with or without the autophagy inhibitor 3-methyladenine (3-MA).

**Figure 6:**
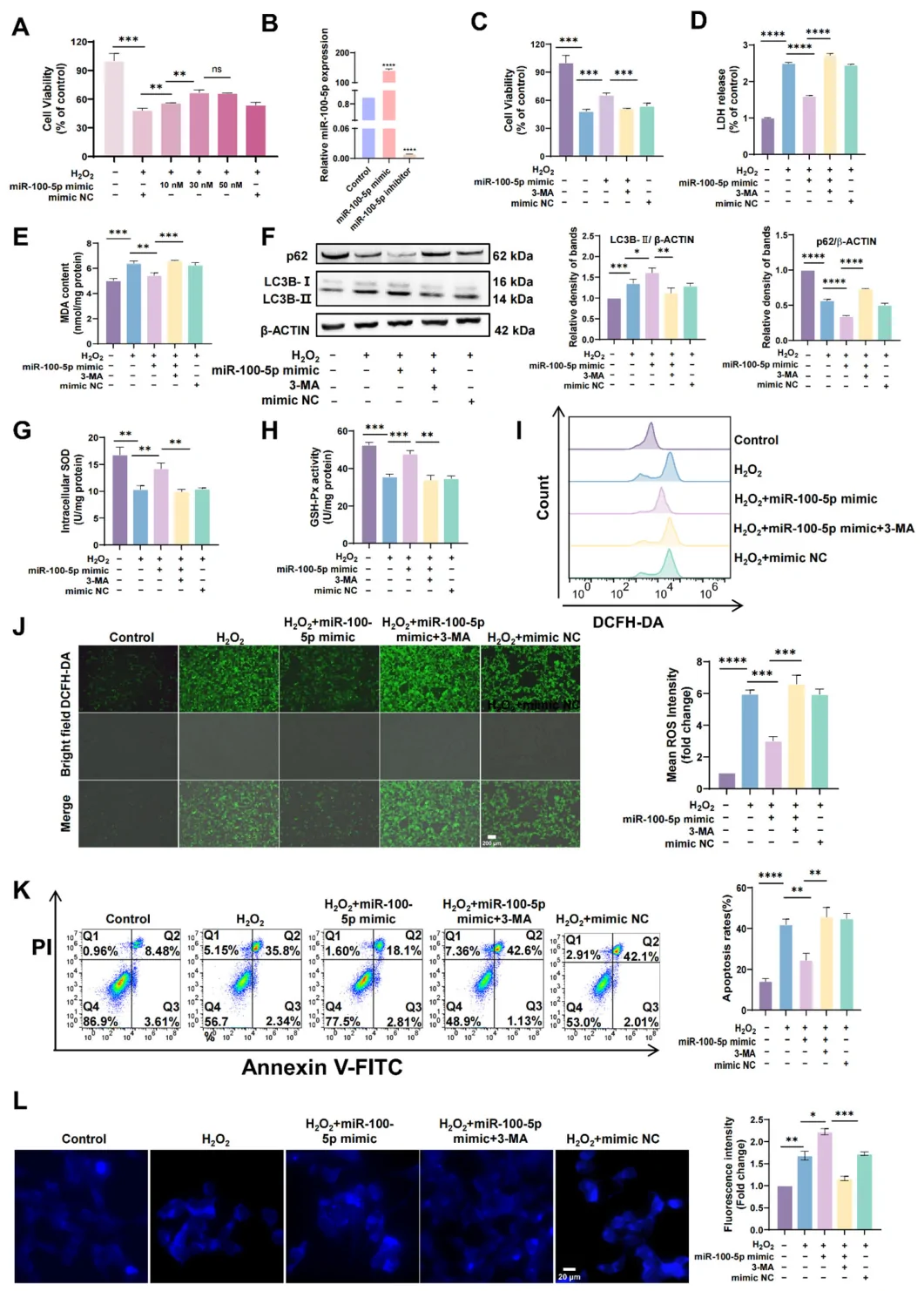
miR-100-5p protects SH-SY5Y cells from H_2_O_2_-induced oxidative injury in an autophagy-dependent manner. (**A**) Determination of the optimal working concentration of the miR-100-5p mimic. SH-SY5Y cells were transfected with gradient concentrations of the miR-100-5p mimic for 24 h, followed by 400 µM H_2_O_2_ stimulation, and cell viability was assessed by CCK-8 assay. (**B**) Validation of transfection efficiency. Intracellular miR-100-5p expression in SH-SY5Y cells transfected with 30 nM miR-100-5p mimic or inhibitor was detected by qPCR. U6 snRNA served as the internal reference for miRNA normalization. (**C**–**L**) SH-SY5Y cells were pretreated as indicated (30 nM miR-100-5p mimic or mimic NC, with or without 4 mM autophagy inhibitor 3-MA) for 24 h, followed by 2 h exposure to 400 µM H_2_O_2_. (**C**) Cell viability detected by CCK-8 assay. (**D**) Lactic dehydrogenase (LDH) release in culture supernatant. (**E**) Intracellular malondialdehyde (MDA) content. (**F**) Western blot analysis of autophagy markers LC3B and p62. β-ACTIN served as the internal loading control. Activities of the antioxidant enzymes: (**G**) superoxide dismutase (SOD) and (**H**) glutathione peroxidase (GSH-Px). (**I**) Quantitative analysis of intracellular reactive oxygen species (ROS) levels by flow cytometry with DCFH-DA staining. (**J**) Representative fluorescence micrographs of intracellular ROS stained with DCFH-DA (green) (scale bar = 200 μm). (**K**) Apoptosis rate of each group, analyzed by flow cytometry with Annexin V-FITC/PI double staining. Dot-plot color gradient: Blue→green→yellow→red represents increasing cell density. (**L**) Representative fluorescence micrographs of intracellular autophagic vacuoles stained with Monodansylcadaverine (MDC) (blue) (scale bar = 20 µm). All quantitative data are presented as the mean ± SD from three independent biological replicates, with five technical replicate wells per sample (**A**,**C**–**E**,**G**,**H**) or three technical replicate wells per sample (**B**,**F**,**I**–**L**) in each experiment. Differences were analyzed by one-way ANOVA followed by Tukey’s post hoc test. * *p* < 0.05; ** *p* < 0.01; *** *p* < 0.001; **** *p* < 0.0001. CCK-8, Cell Counting Kit-8; DCFH-DA, 2’, 7’-dichlorodihydrofluorescein diacetate; NC, negative control.

Transfection efficiency was verified by qPCR: mimic transfection effectively elevated intracellular miR-100-5p levels, whereas inhibitor transfection reduced them (Figure 6B). Based on dose-response analysis, 30 nM mimic was selected for subsequent experiments, as it significantly improved cell viability under H_2_O_2_ challenge (Figure 6A).

We first evaluated the protective effects of miR-100-5p and their dependency on autophagy. Compared with H_2_O_2_ treatment alone, miR-100-5p overexpression significantly increased cell viability (Figure 6C), reduced LDH release (Figure 6D) and MDA content (Figure 6E), enhanced SOD and GSH-Px activities (Figure 6G,H), decreased the apoptosis rate (Figure 6K), and alleviated intracellular ROS accumulation, as demonstrated by fluorescence microscopy and flow cytometry (Figure 6I,J). Co-treatment with 3-MA largely abolished these protective effects: it reversed the improvements in cell viability and apoptosis, attenuated the beneficial effects on plasma membrane integrity and lipid peroxidation, blunted the enhanced antioxidant enzyme activities, and impaired ROS clearance (Figure 6C–E,G–K).

We next examined autophagic activity to confirm its role in miR-100-5p-mediated protection. Western blot analysis showed that the miR-100-5p mimic increased the LC3B-II/β-actin ratio and decreased p62 levels; these changes were reversed by co-treatment with 3-MA (Figure 6F), indicating that miR-100-5p modulates autophagy. Consistently, MDC staining revealed that the increase in autophagic puncta induced by the miR-100-5p mimic was markedly suppressed by 3-MA co-treatment (Figure 6L). Taken together, these results demonstrate that miR-100-5p exerts protective effects against oxidative stress in SH-SY5Y cells via activation of autophagy.

In summary, the present study demonstrates that DPSC-sEVs confer comprehensive protection against oxidative stress, inhibit apoptosis, and improve cell viability in SH-SY5Y cells in a miR-100-5p-dependent manner. Mechanistically, miR-100-5p targets the *MTOR* gene, suppresses its expression, and subsequently activates the cellular autophagy pathway.

## 4. Discussion

Accumulating evidence indicates that DPSC-derived small extracellular vesicles (sEVs) contain abundant bioactive components and exert neuroprotective effects [31,32]. However, the full neuroprotective capacity of DPSC-sEVs and the underlying molecular mechanisms remain largely underexplored. Although miR-100-5p has been shown to regulate mTOR signaling in several tumor models, its role in sEV-mediated neuroprotection has not been established. The present study demonstrates for the first time that DPSC-sEV-associated miR-100-5p suppresses mTOR pathway activity, induces autophagy activation, and attenuates neuronal injury in SH-SY5Y neuronal cells.

Relative to sEVs from other mesenchymal stem cell (MSC) sources, DPSC-derived sEVs possess unique advantages for neural applications. DPSCs originate from the neural crest, endowing their secreted sEVs with inherent neurotropic properties and favorable neuronal tissue compatibility [7]. Additionally, DPSCs can be readily harvested from extracted wisdom teeth or deciduous teeth, providing a non-invasive, abundant and ethically acceptable source of therapeutic sEVs with low immunogenicity. As acellular extracellular vesicles, stem cell-derived sEVs retain the therapeutic merits of parent stem cells while circumventing the biosafety concerns associated with live cell transplantation, including immune rejection and tumor formation [33,34,35,36]. These properties render DPSC-sEVs as a promising cell-free therapeutic candidate for neurodegenerative diseases and acute brain injury [37,38,39].

Characterization of the isolated DPSCs confirmed positive expression of canonical mesenchymal stem cell markers (CD73, CD90, CD105) and near-absence of hematopoietic and immune markers (CD14, CD19, CD34, CD45, HLA-DR). Successful osteogenic and chondrogenic differentiation further verified the multipotent differentiation capacity of DPSCs. The typical cup-shaped morphology, expected particle size distribution, and positive expression of the sEV-specific markers (CD63, CD81, and TSG101) collectively confirmed successful isolation and enrichment of DPSC-sEVs for subsequent functional assays.

Neurons display high metabolic activity and oxygen consumption, and their membranes are enriched in unsaturated fatty acids, rendering them particularly susceptible to oxidative damage. In addition, the antioxidant defense capacity of neural tissues declines progressively with age [40]. Oxidative stress acts as a core pathological driver of a broad spectrum of neurodegenerative disorders and brain injuries, including Alzheimer’s disease, Parkinson’s disease, vascular dementia, and traumatic brain injury [41,42,43]. The SH-SY5Y human neuroblastoma cell line retains key neuronal properties and is widely accepted as a reliable in vitro system for investigating oxidative stress-related neural injury [44,45]. In this study, H_2_O_2_ was used to establish an in vitro model of neuronal oxidative stress. Owing to its chemical stability, high membrane permeability, and ability to recapitulate endogenous oxidative pathological processes, H_2_O_2_ is a standard inducer for establishing standardized neural oxidative injury models [46,47,48].

Our functional experiments showed that pretreatment with DPSC-sEVs significantly improved the viability of SH-SY5Y cells under oxidative stress conditions. DPSC-sEV treatment effectively increased the activities of antioxidant enzymes SOD and GSH-Px and reduced the accumulation of oxidative damage markers, including ROS, LDH, and MDA. Consistent with our results, previous studies have validated the neuroprotective efficacy of DPSC-sEVs in multiple neurological injury models. For example, DPSC-sEVs alleviate neuroinflammation and microglial pyroptosis in subarachnoid hemorrhage [49], ameliorate cerebral ischemia-reperfusion injury by suppressing inflammatory responses [50], and inhibit neuronal apoptosis to promote neurological functional recovery [51].

Although the neuroprotective potential of DPSC-sEVs has been increasingly recognized, the specific effector components within sEVs and their downstream molecular targets underlying these beneficial effects remain largely elusive. sEVs act as vital mediators of intercellular communication by delivering various bioactive cargos. Among these cargos, miRNAs are key regulatory molecules closely involved in the initiation and progression of multiple neurological disorders [52,53,54]. Dysregulated expression of sEV miRNAs contributes to the pathological processes of multiple neurodegenerative diseases, such as Alzheimer’s disease [55,56,57]. miRNAs exert post-transcriptional inhibitory effects by specifically binding to the 3′ untranslated region (3′UTR) of target mRNAs [58]. A limited number of studies have reported that miR-197-3p delivered by DPSC-sEVs attenuates neuroinflammation and microglial pyroptosis via targeting FOXO3 in subarachnoid hemorrhage [49], and that DPSC-sEV-carried miR-877-3p inhibits neuronal apoptosis and facilitates neurological functional recovery via the Bclaf1/p53 pathway [51]. Notably, no study to date has linked DPSC-sEV-carried miR-100-5p to mTOR-mediated autophagy regulation in neuroprotection.

The miR-100-5p/mTOR axis has been well characterized in prior investigations. For instance, miRNA-100 inhibits human bladder urothelial carcinogenesis by directly targeting mTOR [59]. miR-100-5p regulates cardiac hypertrophy by targeting mTOR and consequently activating autophagy [22]. Cao et al. (2023) also reported that miR-100-5p inhibits cerebral infarction progression in mice by suppressing the mTOR pathway to induce autophagy [60]. In addition, miR-100-5p carried by extracellular vesicles from other stem cell sources has been found to exert antioxidant effects against neural injury via the Nox4/ROS/Nrf2 signaling pathway [61,62] and alleviate microglial activation in Parkinson’s disease [63]. Despite these findings, the neuroprotective effects of sEV-carried miR-100-5p via the mTOR-autophagy pathway have not been experimentally established to date.

To delineate the functional role of miR-100-5p in DPSC-sEV-mediated neuroprotection, we transfected SH-SY5Y cells with a specific miR-100-5p inhibitor to block endogenous miR-100-5p function. The results revealed that blockade of miR-100-5p substantially abolished the protective effects of DPSC-sEVs against oxidative injury, indicating that miR-100-5p is an indispensable functional mediator of DPSC-sEV-dependent neuroprotection. Consistent with our data, a recent study demonstrated that miR-100-5p alleviates neuronal oxidative stress in acute cerebral ischemic injury [62]. Collectively, these findings confirm that DPSC-sEVs protect SH-SY5Y cells from H_2_O_2_-induced oxidative damage in a miR-100-5p-dependent manner.

Bioinformatic analysis predicted *MTOR* as a direct downstream target of miR-100-5p, with highly conserved binding sites in its 3′UTR. The mTOR kinase is a central negative regulator of autophagy: activation of mTOR suppresses autophagy, whereas inhibition of mTOR triggers autophagy induction [64,65]. Autophagy is an essential endogenous protective mechanism that eliminates damaged organelles and oxidized proteins under stress, thereby limiting oxidative stress-induced neuronal apoptosis [66]. We found that miR-100-5p directly targets the *MTOR* 3′UTR; however, the reduction of total mTOR protein was moderate, whereas phosphorylated mTOR (p-mTOR) was significantly downregulated.

This discrepancy can be accounted for by several mechanisms. First, miRNA-mediated regulation of *MTOR* occurs primarily through translational repression rather than robust mRNA degradation. Given the long half-life of mTOR protein, inhibition of de novo synthesis only gradually reduces the total protein pool, resulting in a mild downward trend. Second, the phosphorylated active mTOR pool has a faster turnover rate and depends on continuous new protein synthesis for complex assembly and activation, making it more sensitive to translational inhibition. Third, miR-100-5p may also target upstream activators of the mTOR pathway, further suppressing mTOR phosphorylation. Together, these effects lead to a more pronounced reduction in active p-mTOR, which ultimately mediates the biological function of miR-100-5p in DPSCs. Functional experiments showed that both DPSC-sEV treatment and miR-100-5p mimic transfection upregulated LC3B-II expression and reduced p62 accumulation, accompanied by alleviated oxidative damage and improved cell viability. In contrast, pharmacological inhibition of autophagy using 3-MA markedly reversed the protective effects of DPSC-sEVs. These findings suggest that miR-100-5p exerts neuroprotective and antioxidant effects, at least in part, through modulation of autophagy via mTOR suppression.

Appropriate activation of autophagy facilitates the clearance of intracellular toxic metabolites and damaged organelles, maintains cellular homeostasis, and enhances neuronal survival under stress [67]. Accumulating evidence has verified that moderate autophagy activation alleviates neuronal damage in various neurological disease models. Proper autophagy reduces excessive ROS accumulation and stabilizes intracellular energy metabolism in the early stage of cerebral ischemia [68], promotes neurological functional recovery after spinal cord injury [69], accelerates nerve regeneration [70,71], slows the progression of neurodegenerative diseases [72], and exerts neuroprotective effects [73]. Our results are consistent with these previous studies, showing that targeted autophagy activation mitigates neuronal apoptosis, ROS generation, and lipid peroxidation under oxidative stress.

Notably, autophagy exerts a dual effect on neuronal survival. Either insufficient or excessive autophagy disrupts the homeostasis of intracellular protein and organelle turnover, ultimately leading to neuronal death [74]. Excessively activated autophagy causes aberrant self-degradation of essential cellular organelles and proteins, exacerbating neuronal injury in the late phase of cerebral ischemia [75]. In addition, dysfunction of the autophagy-lysosomal system contributes to toxic protein aggregation in multiple neurodegenerative disorders [76]. Therefore, precise modulation of autophagy homeostasis is essential for optimal neuroprotective outcomes.

In summary, this study identifies and validates miR-100-5p as a key functional cargo of DPSC-sEVs and reveals a novel neuroprotective mechanism mediated by the miR-100-5p/mTOR/autophagy signaling axis. Previous studies on DPSC-sEV-mediated neural protection have mainly focused on miR-197-3p [49] and miR-877-3p [51]. Our findings expand the functional repertoire of DPSC-sEV-derived miRNAs and provide a novel molecular basis for the antioxidant and anti-apoptotic properties of DPSC-sEVs in neural injury.

Several limitations of this study should be acknowledged. First, sEVs were isolated via PEG precipitation, a method that enriches rather than purifies sEVs and may co-isolate soluble proteins, protein aggregates, and lipoproteins. Furthermore, negative markers of non-vesicular contaminants were not included in our characterization panel, so we cannot definitively exclude contamination from intracellular compartments or non-vesicular factors. Accordingly, the observed biological effects should be attributed to sEV-enriched fractions rather than pure sEV preparations. Second, all mechanistic experiments were conducted exclusively in the SH-SY5Y neuroblastoma cell line. The use of a single cell system restricts the generalizability of our findings. Further validation in additional neuronal cell lines and primary neuron cultures will be important to confirm the biological relevance of the miR-100-5p/mTOR regulatory axis identified here. Third, all functional and mechanistic assays were performed in vitro, without further validation in animal models. This simplified in vitro system lacks the multicellular crosstalk, extracellular matrix, blood supply and inflammatory components present in intact neural tissue. Differences between cell culture and native tissue microenvironments may influence the severity of oxidative injury and the biological activity of DPSC-sEVs. The acute H_2_O_2_-induced injury model used here represents a simplified form of oxidative insult and cannot fully recapitulate the complex pathological processes of chronic neurodegenerative diseases. Further studies using ex vivo nerve tissue or in vivo peripheral neuronal injury models are required to verify the translational relevance of our findings.

In addition to the aforementioned limitations of the in vitro system, another important consideration is the physiological relevance of the sEV miR-100-5p dosage used in this study. In our in vitro system, the effective dose of DPSC-sEVs falls within the typical range reported in sEV-based neural protection studies. Nevertheless, the in vivo physiological concentration of miR-100-5p in peripheral nerve tissue and its dynamic changes during injury and repair have not been fully characterized in the present study. Native sEVs exhibit rapid clearance and low nerve targeting, which limits the bioavailability of miR-100-5p at lesion sites. Potential strategies to address this include miR-100-5p enrichment via parental cell engineering, surface modification with nerve-homing peptides, and local perineural delivery, all of which requiring further preclinical validation.

## Figures and Tables

**Table 1:** Specific primer sequences for qPCR.

Gene	Species	Direction	Primer Sequence 5′→3′
U6	Human	Forward	CTCGCTTCGGCAGCACA
		Reverse	AACGCTTCACGAATTTGCGT
miR-7-5p	Human	Reverse Transcription	GTCGTATCCAGTGCAGGGTCCGAGGTATTCGCACTGGATACGACAACAAC
		Forward	CGCGTGGAAGACTAGTGATTTT
		Reverse	AGTGCAGGGTCCGAGGTATT
miR-21-5p	Human	Reverse Transcription	GTCGTATCCAGTGCAGGGTCCGAGGTATTCGCACTGGATACGACTCAACA
		Forward	GCGCGTAGCTTATCAGACTGA
		Reverse	AGTGCAGGGTCCGAGGTATT
miR-31-5p	Human	Reverse Transcription	GTCGTATCCAGTGCAGGGTCCGAGGTATTCGCACTGGATACGACAGCTAT
		Forward	GCGAGGCAAGATGCTGGC
		Reverse	AGTGCAGGGTCCGAGGTATT
miR-100-5p	Human	Reverse Transcription	GTCGTATCCAGTGCAGGGTCCGAGGTATTCGCACTGGATACGACCACAAG
		Forward	GCGAACCCGTAGATCCGAA
		Reverse	AGTGCAGGGTCCGAGGTATT
miR-1246	Human	Reverse Transcription	GTCGTATCCAGTGCAGGGTCCGAGGTATTCGCACTGGATACGACCCTGCT
		Forward	GCGCGAATGGATTTTTGG
		Reverse	AGTGCAGGGTCCGAGGTATT

**Table 2:** Sequences of synthetic miRNA oligonucleotides.

Oligonucleotide	Sequence (5′→3′)
miR-100-5p mimic sense	AACCCGUAGAUCCGAACUUGUG
miR-100-5p mimic antisense	CACAAGUUCGGAUCUACGGGUU
Mimic negative control sense	UUCUCCGAACGUGUCACGUTT
Mimic negative control antisense	ACGUGACACGUUCGGAGAATT
miR-100-5p inhibitor	CACAAGUUCGGAUCUACGGGUU
Inhibitor negative control	CAGUACUUUUGUGUAGUACAA

**Table 3:** Profiling of miRNAs in DPSC-sEVs [19].

Category	miRNAs
miRNAs detected in DPSC-sEVs	miR-22, miR-27a, miR-30b-5p, miR-324-5p, miR-130a-3p, miR-34a-5p, miR-378f, miR-513b-5p, miR-193a-5p, miR-5100, miR-4792, miR-652-3p, miR-505-3p, miR-27a-5p, miR-629-5p, miR-1260b, miR-140-3p, miR-1260a, miR-185-5p, miR-146b-5p, miR-339-5p, miR-1246, miR-107, miR-370-3p, miR-320d, miR-210-3p, miR-451a, miR-10a-5p, miR-215-5p, miR-1-3p, miR-126-3p, miR-10b-5p, miR-3687, miR-619-5p, miR-31-5p, miR-370-3p, miR-505-3p, miR-451a, let-7f-1-3p, miR-193a-5p, miR-4792, miR-3687
The five most abundant miRNAs in DPSC-sEVs	miR-31, miR-21-5p, miR-100-5p, miR-1246, miR-7b-5p

**Table 4:** Partial target genes of miR-100-5p.

miRNA Name	Target Gene Name
miR-100-5p	*MTOR*, *EPDR1*, *HS3ST2*, *KBTBD8*, *FKBP5*, *SMARCA5*, *FZD8*, *RASGRP3*, *CLDN11*, *TRIB2*, *ZBTB7A*, *TMEM135*, *ST5*, *PPP1CB*, *NXF1*, *SLC44A1*, *RRAGD*, *ZZEF1*, *CYP26B1*, *TAOK1*, *THAP2*, *BMPR2*, *ZNRF2*, *AGO2*, *FZD5*

## Data Availability

The data that support the findings of this study are available from the corresponding author upon reasonable request.

## Supplementary Materials

The following supporting information can be downloaded at: https://www.mdpi.com/article/10.3390/cells15201843/s1, Figure S1: Effects of miR-21-5p and miR-31-5p mimics on the viability of SH-SY5Y cells under H_2_O_2_-induced oxidative injury. SH-SY5Y cells were transfected with 30 nM, 50 nM, and 100 nM miR-21-5p mimic (A) or miR-31-5p mimic (B); mimic negative control (NC) served as the transfection control. Subsequently, cells were exposed to 400 µM H_2_O_2_, and cell viability was assessed via the CCK-8 assay. All quantitative data are presented as the mean ± SD from three independent biological replicates, with triplicate technical replicates per experiment. Differences were analyzed by one-way ANOVA followed by Tukey’s post hoc test. *** *p* < 0.001; ns, no significant difference. CCK-8, Cell Counting Kit-8; miR, microRNA; mimic, microRNA mimic; NC, negative control.

## Abbreviations

The following abbreviations are used in this manuscript:

DPSCs	dental pulp stem cells
DPSC-sEVs	DPSC-derived sEVs
DLS	dynamic light scattering
GSH-Px	glutathione peroxidase
H_2_O_2_	hydrogen peroxide
LDH	lactate dehydrogenase
miRNAs	microRNAs
MSCs	mesenchymal stem cells
mTOR	mammalian target of rapamycin
MDA	malondialdehyde
ROS	reactive oxygen species
sEVs	small extracellular vesicles
SOD	superoxide dismutase
TEM	transmission electron microscopy
