# Supplementary material for: Dental Pulp Stem Cell-Derived Small Extracellular Vesicle miR-100-5p Attenuates Neuronal Oxidative Stress via Regulating the mTOR-Autophagy Signaling Axis
Source: Cells. 2026 Oct 11;15(20):1843. doi: 10.3390/cells15201843 (PMC13702593; doi:10.3390/cells15201843)
Supplement: Supplementary file 1 [file cells-15-01843-s001.zip › cells-4550068-supplementary.pdf]

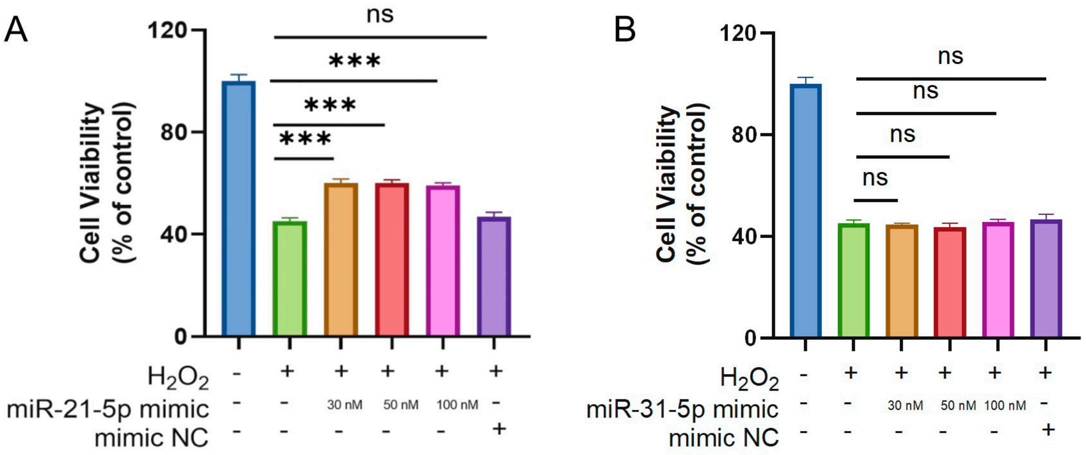

Figure S1. Effects of miR-21-5p and miR-31-5p mimics on the viability of SH-SY5Y cells under H_2O_2-induced oxidative injury. SH-SY5Y cells were transfected with 30 nM, 50 nM, and 100 nM miR-21-5p mimic (A) or miR-31-5p mimic (B); mimic negative control (NC) served as the transfection control. Subsequently, cells were exposed to 400 μM H_2O_2, and cell viability was assessed via the CCK-8 assay. All quantitative data are presented as the mean ± SD from three independent biological replicates, with triplicate technical replicates per experiment. Differences were analyzed by one-way ANOVA followed by Tukey's post hoc test. ***$p < 0.001$; ns, no significant difference. CCK-8, Cell Counting Kit-8; miR, microRNA; mimic, microRNA mimic; NC, negative control.
